# Asymmetric N-Cadherin Expression Results in Synapse Dysfunction, Synapse Elimination, and Axon Retraction in Cultured Mouse Neurons

**DOI:** 10.1371/journal.pone.0054105

**Published:** 2013-01-31

**Authors:** Kim N. Pielarski, Bernd van Stegen, Aksana Andreyeva, Katja Nieweg, Kay Jüngling, Christoph Redies, Kurt Gottmann

**Affiliations:** 1 Institute of Neuro- and Sensory Physiology, Medical Faculty, Heinrich-Heine-University Düsseldorf, Düsseldorf, Germany; 2 Institute of Anatomy I, University of Jena School of Medicine, Jena University Hospital, Jena, Germany; Institute for Interdisciplinary Neuroscience, France

## Abstract

Synapse elimination and pruning of axon collaterals are crucial developmental events in the refinement of neuronal circuits. While a control of synapse formation by adhesion molecules is well established, the involvement of adhesion molecules in developmental synapse loss is poorly characterized. To investigate the consequences of mis-match expression of a homophilic synaptic adhesion molecule, we analysed an asymmetric, exclusively postsynaptic expression of N-cadherin. This was induced by transfecting individual neurons in cultures of N-cadherin knockout mouse neurons with a N-cadherin expression vector. 2 days after transfection, patch-clamp analysis of AMPA receptor-mediated miniature postsynaptic currents revealed an impaired synaptic function without a reduction in the number of presynaptic vesicle clusters. Long-term asymmetric expression of N-cadherin for 8 days subsequently led to synapse elimination as indicated by a loss of colocalization of presynaptic vesicles and postsynaptic PSD95 protein. We further studied long-term asymmetric N-cadherin expression by conditional, Cre-induced knockout of N-cadherin in individual neurons in cultures of N-cadherin expressing cortical mouse neurons. This resulted in a strong retraction of axonal processes in individual neurons that lacked N-cadherin protein. Moreover, an *in vivo* asymmetric expression of N-cadherin in the developmentally transient cortico-tectal projection was indicated by in-situ hybridization with layer V neurons lacking N-cadherin expression. Thus, mis-match expression of N-cadherin might contribute to selective synaptic connectivity.

## Introduction

During the development of the mammalian nervous system, early axon outgrowth and initial synaptogenesis are followed by refinement of neuronal circuits involving both elimination of synapses and pruning of exuberant axon collaterals [1], [2], [3], [4]. Among other molecular players, cell adhesion molecules are thought to be involved in the control of axon guidance and synapse formation [5], [6], [7], [8], [9], [10]. However, their roles in synapse elimination and axon retraction are less well understood. The synaptic adhesion molecule N-cadherin has been described to be required for the formation and early maturation of synapses in immature neurons. Both presynaptic vesicle accumulation and postsynaptic spine formation are dependent on N-cadherin function [11], [12], [13], [14], [15], [16], [17], [18], [19].

N-cadherin is well known to be symmetrically expressed in the pre- and the postsynaptic membrane of mature glutamatergic synapses [20], [21], [22], [23], [24]. It mediates transsynaptic adhesion by homophilic interaction of the extracellular domains across the synaptic cleft. On the cytoplasmic side N-cadherin is associated with several types of catenins thus enabling signaling into the pre- and postsynaptic compartments [25], [26], [27] Although N-cadherin has been described to be strongly involved in synaptogenesis, the complete loss of N-cadherin in N-cadherin knockout neurons and mice is well compensated resulting in an unaltered synapse number and maintenance in mature N-cadherin deficient neurons [19], [28], [29]. *In vivo*, functionally connected neurons and brain areas have been described to express matching subtypes of cadherins [30], [31], [32], [33], [34], further demonstrating the physiological importance of homophilic cadherin interactions.

In addition to this classical symmetric expression of cadherins on both sides of synapses, it is conceivable that N-cadherin might be expressed exclusively pre- or postsynaptically during developmental refinement processes thus leading at least transiently to mis-match situations. Previously, we had started to investigate the functional consequences of an asymmetric expression of N-cadherin in cocultures of N-cadherin knockout neurons and N-cadherin expressing cortical neurons [28]. As studied by paired patch-clamp recordings, exclusively presynaptic expression of N-cadherin resulted in a relatively weak phenotype, i.e. an enhanced synaptic depression upon repetitive stimulation, which was similar to the complete pre- and postsynaptic knockout of N-cadherin [28]. However, the exclusively postsynaptic expression of N-cadherin resulted in a surprisingly strong phenotype, i.e. reduced amplitudes and increased failure rates of AMPA EPSCs elicited by single action potentials, indicating a strong functional defect [28].

In this paper, we have now addressed whether the absence of N-cadherin on only the presynaptic side of the synapse, i.e. an exclusively postsynaptic N-cadherin expression, affects the long-term stability of synaptic connections. We studied the consequences of this asymmetric N-cadherin expression using a well controlled *in vitro* approach consisting of N-cadherin expression in individual N-cadherin knockout neurons. We show that an asymmetric, exclusively postsynaptic expression of N-cadherin leads to impaired synaptic function, synapse elimination and axon retraction. N-cadherin mis-match expression might be of importance in developmentally transient connections such as the cortico-tectal projection of the somatosensory system.

## Results

### Asymmetric, selectively postsynaptic expression of N-cadherin impairs synaptic function

**Figure 1:**
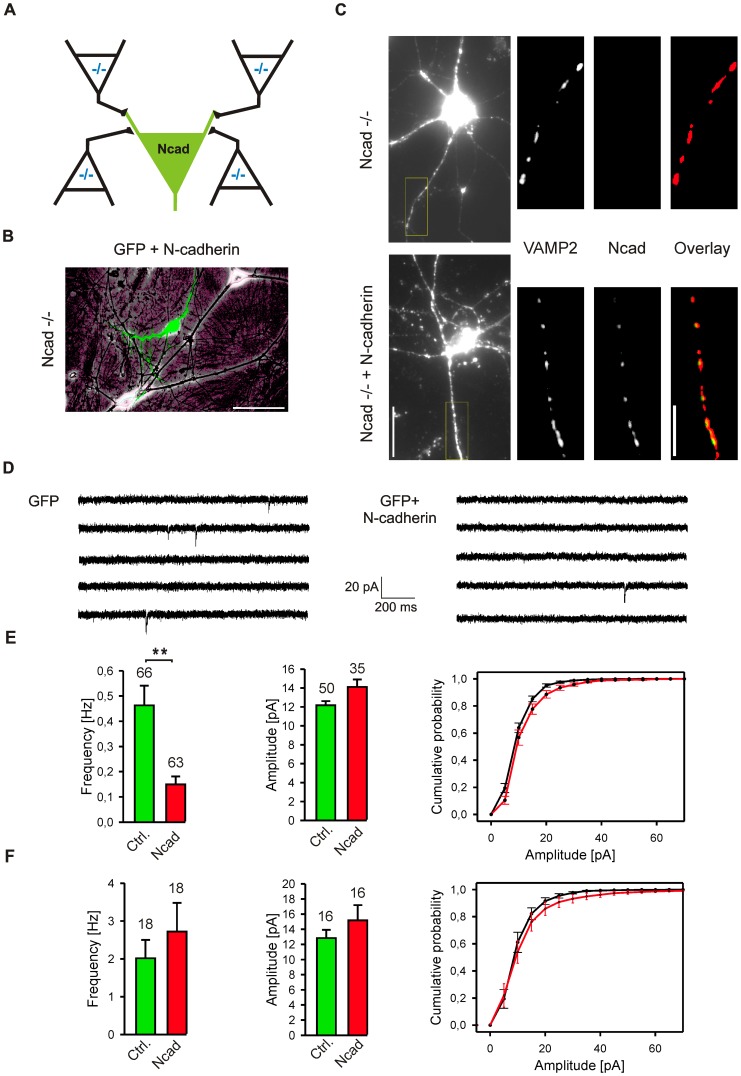
Asymmetric, selectively postsynaptic expression of N-cadherin impairs basal function of glutamatergic synapses. (**A**) Scheme of N-cadherin+EGFP expression in individual, transfected N-cadherin knockout neurons (Ncad; green) innervated by surrounding, not transfected N-cadherin knockout neurons (−/−; derived from mouse ES cells). (**B**) Overlay of a phase contrast image of cultured N-cadherin knockout neurons and the corresponding fluorescence image of a N-cadherin+EGFP transfected neuron (12 DIV, 2 days after transfection). Scale bar represents 30 µm. (**C**) *Left:* Fluorescence images (VAMP2 immunostaining) of a knockout (Ncad−/−) and a transfected (Ncad−/− + N-cadherin) neuron immunostained for VAMP2 and N-cadherin. The dendritic segment indicated in the whole cell image is shown enlarged and thresholded. *Center:* Corresponding N-cadherin immunostaining of the same dendritic segment. *Right:* Overlay of VAMP2 and N-cadherin immunostaining of the same dendritic segment. Scale bars represent 15 µm and 5 µm. (**D, E**) Spontaneous, AMPA receptor mediated miniature EPSCs recorded from an EGFP transfected, N-cadherin knockout neuron (GFP, control) and from a N-cadherin+EGFP transfected, N-cadherin knockout neuron (Ncad, mis-match). Patch-clamp recordings 2 days after transfection; holding potential −60 mV. (**E**) Quantification of the frequency and the amplitude (cells without mEPSCs not included) of AMPA mEPSCs (11–13 DIV). Cumulative distribution of mEPSC amplitudes is shown in the right panel. Note the strong reduction in mini frequency in neurons with asymmetric N-cadherin expression. (**F**) Control postsynaptic overexpression of N-cadherin in primary cultured cortical neurons. Patch-clamp recordings 2 days after transfection; holding potential −60 mV. Quantification of the frequency and the amplitude of AMPA mEPSCs with cumulative distribution of mEPSC amplitudes shown in the right panel. Means ± SEM. *n* (cells) is indicated on bars. **, *P*<0.001 Students t-test.

To investigate the consequences of an asymmetric expression of N-cadherin, i.e. knockout in the presynaptic neurons and expression in an individual postsynaptic neuron, we expressed N-cadherin in individual cultured cells, which were innervated by N-cadherin knockout neurons (Fig. 1A; Fig. S1A). This culture system was based on N-cadherin knockout, ES cell derived neurons that were in vitro differentiated from homozygous knockout ES cells [28], [35]. Mouse ES cell derived neurons exhibit functional synapses with properties similar to primary cultured cortical neurons [36], [37], [38]. N-cadherin expression in individual, ES cell-derived knockout neurons was induced by low efficiency Lipofectamine transfection of an N-cadherin expression vector (cotransfection with EGFP, Fig. 1B) at 9–11 DIV after initial synapse formation had occurred. Control cells were transfected with EGFP vector only. The functionality of the N-cadherin construct was checked by a cell aggregation assay (Fig. S1B). As studied by EGFP fluorescence imaging, 2 days after transfection the morphology of N-cadherin expressing cells was similar to controls. Quantitative analysis of the total dendritic branch length revealed no significant differences (Fig. S1D).

To confirm the expression of N-cadherin in transfected neurons, immunocytochemical costainings for N-cadherin and for the vesicle marker VAMP2 to identify synaptic sites on dendrites were performed 2 days after transfection. In N-cadherin knockout neurons, a punctate VAMP2 staining along dendrites was obtained, whereas no specific N-cadherin staining was detectable (Fig. 1C). This punctate VAMP2 pattern is typical for presynaptic vesicle clusters in primary cultured neurons. In N-cadherin expressing neurons, a similar punctate VAMP2 staining was found, and dendritic N-cadherin puncta were present in addition. The dendritic density of N-cadherin puncta was slightly lower as compared to the dendritic density of N-cadherin puncta in primary cultured cortical neurons (Fig. S1C) indicating that N-cadherin was expressed at a physiological level. N-cadherin puncta were colocalized with VAMP2 puncta, demonstrating the synaptic targeting of expressed N-cadherin (Fig. 1C). Quantitatively, the vast majority of N-cadherin puncta colocalized with VAMP2 puncta (93±4%, *n* = 24 cells), while only a fraction of the VAMP2 puncta colocalized with N-cadherin (71±5%) most likely because of the additional presence of GABAergic synapses devoid of N-cadherin [21].

After having established an asymmetric N-cadherin expression, we first addressed, whether the function of glutamatergic synapses is affected in this N-cadherin mis-match situation. We focused on glutamatergic synapses, because N-cadherin has been described to be restricted to glutamatergic synapses in mature cultured neurons [21]. Spontaneous, AMPA receptor-mediated miniature postsynaptic currents (AMPA mEPSCs) were recorded in the presence of TTX from transfected neurons using the whole-cell patch-clamp technique. Strikingly, 2 days after transfection the frequency of AMPA mEPSCs was drastically (*P*<0.001) reduced in N-cadherin expressing neurons (0.15±0.03 Hz) as compared to control EGFP expressing neurons (0.46±0.08 Hz; Fig. 1D, E). The mean amplitudes of AMPA mEPSCs were not significantly affected by asymmetric N-cadherin expression. Analysis of cumulative distributions of AMPA mEPSC amplitudes revealed a slight trend to increased amplitudes upon N-cadherin expression (Fig. 1E). We further performed control expression of N-cadherin in individual primary cultured cortical neurons endogeneously expressing N-cadherin (Fig. 1F). In this N-cadherin overexpression situation without a strong mis-match, AMPA mEPSC frequency was not significantly affected confirming that a strong mis-match expression is required for induction of a functional synaptic defect. Analysis of cumulative distributions of AMPA mEPSC amplitudes revealed again a slight trend to increased amplitudes upon N-cadherin expression (Fig. 1F). This might point to a direct cis-interaction of postsynaptically expressed N-cadherin and AMPA receptors [39]. Taken together, our findings indicate that the function and/or the maintenance of glutamatergic synapses is impaired in the N-cadherin mis-match situation.

In our culture system neurons form not only synapses, but to a relatively low extent also autapses, which express N-cadherin both pre- and postsynaptically upon transfection of an N-cadherin vector. To study the effects of N-cadherin expression on autapses, whole-cell patch-clamp recordings were obtained from transfected neurons. Two days after transfection, action potentials were elicited by activating somatic Na^+^ currents with short depolarizing pulses, and autaptic AMPA receptor-mediated EPSCs were recorded (Fig. 3A). About 60% of the recorded neurons did not exhibit any autaptic PSCs, although the extracellular Ca^2+^ concentration had been elevated from 2,5 mM to 5 mM. In the remaining neurons neither the mean amplitude nor the mean failure rate of autaptic AMPA EPSCs was significantly different between N-cadherin expressing and control neurons (Fig. 3B). These results again indicate that a synaptic mis-match of N-cadherin is required to induce an impairment of synaptic function upon N-cadherin expression. In summary, the observed strong reduction in AMPA mEPSC frequency might be explained either by a functional defect or by a complete loss of synapses. To clarify this point, a potential contribution of a reduced synapse number was further investigated.

### Long-term asymmetric expression of N-cadherin induces synapse elimination

**Figure 2:**
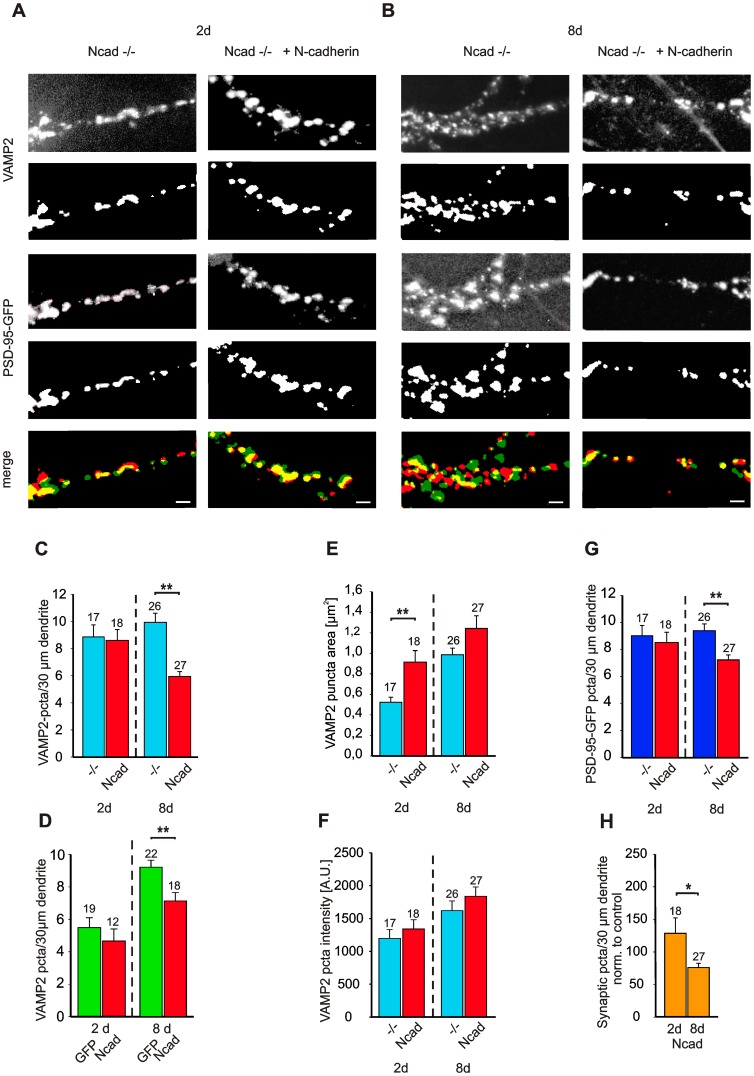
Long-term asymmetric expression of N-cadherin induces elimination of glutamatergic synapses. (**A, B**) Immunocytochemical stainings for VAMP2 and fluorescence images of coexpressed PSD-95-EGFP in N-cadherin knockout neurons (Ncad−/−) and in N-cadherin knockout neurons expressing N-cadherin (Ncad−/− + N-cadherin) 2 days (A) and 8 days (B) after transfection at 14 DIV. Segments of proximal dendrites are shown. Scale bars: 2,5 µm. Upper panels are fluorescence images and lower panels are thresholded (black or white) images used to quantify puncta. Dendrites were identified using the PSD-95-EGFP images. Merge: Overlay of thresholded VAMP2 (red) and PSD-95-EGFP (green) puncta; >80% of PSD-95 puncta colocalized with VAMP2. (**C, E, F**) Quantification of dendritic density of VAMP2 puncta (including few autaptic puncta, C), VAMP2 puncta area (E), and VAMP2 puncta average intensity (F). −/−: N-cadherin knockout neurons; Ncad: N-cadherin knockout neurons expressing N-cadherin. (**G**) Quantification of dendritic density of PSD-95-EGFP puncta. (**H**) Relative changes of dendritic density of glutamatergic synapses (colocalized VAMP2 and PSD-95-EGFP puncta, excluding autaptic puncta) in N-cadherin knockout neurons expressing N-cadherin. Normalized to values of N-cadherin knockout neurons (control) at matched times in culture. Note that selectively at 8 days after transfection (8d, 22 DIV), but not at 2 days after transfection (2d, 16 DIV), the dendritic density of synapses is reduced. (**D**) Quantification of dendritic density of VAMP2 puncta 2 days (2d) and 8 days (8d) after transfection at an earlier stage in culture (9–11 DIV). GFP: control N-cadherin knockout neurons. Ncad: N-cadherin knockout neurons expressing N-cadherin. Note the reduced increase in synapse density in N-cadherin knockout neurons expressing N-cadherin at 8 days after transfection. Means ± SEM. *n* (cells) is indicated on bars. *, *P*<0.05; **, *P*<0.01 and *P*<0.001 (in C, G) Students t-test.

To analyse changes in synapse number, we immunocytochemically stained presynaptic vesicle clusters for VAMP2 and determined the density of VAMP2 puncta on the EGFP-labeled dendrites of N-cadherin expressing (cotransfection with EGFP) and control (transfection with only EGFP) ES cell derived neurons. 2 days after transfection (at 9–11 DIV) the dendritic density of VAMP2 puncta did not significantly differ between N-cadherin expressing and control neurons (Fig. 2D). This indicates that the observed reduction in AMPA mEPSC frequency is primarily caused by a functional synaptic defect. However, 8 days after transfection a significant (*P*<0.01) reduction in the dendritic density of VAMP2 puncta was found upon long-term asymmetric expression of N-cadherin (Fig. 2D). Because effects on synapse numbers were studied in this initial experiment during the major phase of in vitro synaptogenesis (first two weeks), this finding might be explained by reduced synapse formation as well as elimination of already existing synapses.

To demonstrate that asymmetric N-cadherin expression induces elimination of synapses, we transfected ES cell derived neurons at 14 DIV after the steepest increase in synapse numbers had occurred. N-cadherin was coexpressed together with PSD-95-EGFP (to label postsynaptic sites) and DsRed2-VAMP2 (to identify autapses). In control cells PSD-95-EGFP and DsRed2-VAMP2 were coexpressed without N-cadherin. In addition, transfected cultures were immunostained for VAMP2 to label all presynaptic vesicle clusters (including the few autaptic contacts formed). (Fig. 2). 2 days after transfection, neither the dendritic density of immunostained VAMP2 puncta (Fig. 2C) nor the dendritic density of PSD-95-EGFP puncta (Fig. 2G) was significantly altered. Seemingly counter-intuitively to the strongly reduced AMPA mEPSC frequency (Fig. 1), VAMP2 puncta area was significantly (*P*<0,01) increased 2 days after transfection (Fig. 2E), while VAMP2 puncta intensity was unchanged (Fig. 2F). Because a functional compensation of synaptic inactivity by an increase in presynaptic vesicle cluster size is well known from previous reports in cultured hippocampal neurons [40], [41], this finding indicates the presence of a compensatory mechanism that however can only partially balance the above described impaired synaptic function.

**Figure 3:**
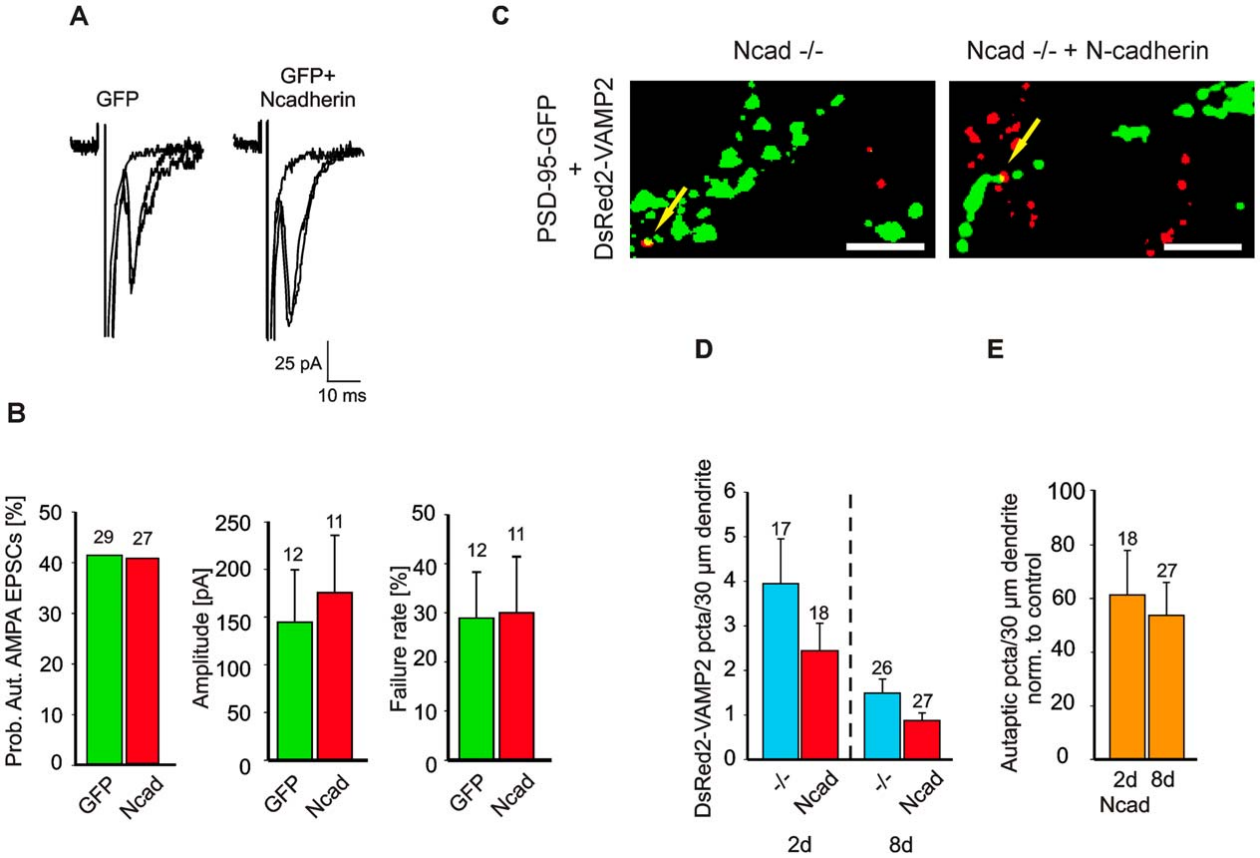
Symmetric, pre- and postsynaptic expression of N-cadherin at autapses does not impair synaptic function. (**A**) Autaptic AMPA EPSCs recorded from N-cadherin knockout neurons transfected with either EGFP (GFP) or N-cadherin+EGFP (GFP+N-cadherin, Ncad). Autaptic AMPA EPSCs were elicited by action currents induced by depolarizing pulses in the same neuron. Holding potential −60 mV. 3 traces superimposed. Stimulation artefacts and Na^+^ currents are truncated. (**B**) Quantification of autaptic AMPA EPSCs. *Left:* Probability of occurrence of autaptic AMPA EPSCs (5mM Ca^2+^). 11–13 DIV, 2 days after transfection. *Center:* Peak amplitudes. *Right:* Failure rates. (**C, D, E**) Incidence of autapses is not significantly affected in N-cadherin knockout neurons expressing N-cadherin. (**C**) Autapses were identified by coexpression of DsRed2-VAMP2 and PSD-95-EGFP in N-cadherin knockout neurons (Ncad−/−, control) and in N-cadherin knockout neurons expressing N-cadherin (Ncad−/− + N-cadherin). Overlays of corresponding DsRed2-VAMP2 (red) and PSD-95-EGFP (green) fluorescence images (8 days after transfection; 22 DIV, same cells as in Fig. 2B) that were strongly thresholded to visualize the rare autapses (arrows). Scale bars: 2,5 µm. (**D**) Quantification of dendritic density of DsRed2-VAMP2 puncta (autapses) 2 days (2d) and 8 days (8d) after transfection at 14 DIV. −/−: N-cadherin knockout neurons. Ncad: N-cadherin knockout neurons expressing N-cadherin. Same cells as in Fig. 2. Note the strong (non-significant) trend to a general reduction in the number of autapses with time in culture. (**E**) No significant changes in the dendritic density of glutamatergic autapses (colocalized DsRed2-VAMP2 puncta and PSD-95-EGFP puncta) were induced by N-cadherin expression (Ncad). Normalized to values of N-cadherin knockout neurons (control) at matched times in culture. Means ± SEM. *n* (cells) is indicated on bars. Students t-test, ANOVA (D).

Strikingly, 8 days after transfection upon long-term expression of N-cadherin, the dendritic density of VAMP2 puncta (5,9±0,4 per 30µm dendrite) was significantly (*P*<0,001) reduced as compared to controls (9,9±0,7 per 30µm dendrite; Fig. 2C). VAMP2 puncta area and intensity were not significantly affected. Similarly, the dendritic density of PSD-95-EGFP puncta was significantly (*P*<0,001) reduced at 8 days after transfection (Fig. 2G). Most importantly, upon long-term asymmetric expression of N-cadherin both the dendritic density of VAMP2 puncta and that of PSD-95-EGFP puncta at 22 DIV were clearly lower than at 16 DIV demonstrating the elimination of synapses (Fig. 2C, G). To quantify the extent of synapse elimination, colocalized VAMP2 (now excluding autaptic, DsRed2-VAMP2 labeled sites) and PSD-95-EGFP puncta were counted. 8 days after transfection the dendritic density of glutamatergic synapses was significantly (*P*<0.05) reduced to 76,1±6,6% of controls (Fig. 2H). The number of autapses (DsRed2-VAMP2 puncta) was highly variable and showed a strong, but statistically not significant trend to decrease with time in culture in both control and N-cadherin expressing neurons with very few autapses detectable at 22 DIV (Fig. 3C–E). Despite a slight trend, the number of autapses was not significantly affected at both 2 and 8 days after N-cadherin transfection. In summary, our results demonstrate that a long-term asymmetric expression of N-cadherin induces synapse elimination, which appeared to be preceeded by an impairment of synaptic function.

### Long-term asymmetric expression of N-cadherin induces axon retraction

During development, synapse elimination is accompanied by retraction of axonal branches thus strongly contributing to selective synaptic connectivity [2], [4]. To address whether long-term asymmetric expression of N-cadherin also leads to axon retraction, we induced a conditional N-cadherin knockout in individual neurons resulting in presynaptic N-cadherin knockout in individual axons innervating N-cadherin expressing postsynaptic neurons. In cultures of cortical neurons from homozygous floxed N-cadherin mice [42], individual neurons were transfected with a creEGFP expression vector (low-efficiency Lipofectamine technique) [43]. Cotransfection with EGFP was done to visualize the dendrites and the axon of the transfected neurons. To verify the loss of N-cadherin in individual creEGFP transfected neurons at the protein level, cultures were immunocytochemically stained with antibodies against N-cadherin and the expression of N-cadherin protein was analysed in transfected neurons as described by [29]. 2 days after transfection (at 5 DIV), individual EGFP expressing control neurons showed a strong expression of N-cadherin (Fig. S2A). Individual CreEGFP+EGFP expressing neurons exhibited – after this short time period – a similar expression of N-cadherin suggesting that N-cadherin protein is still present despite the Cre-induced gene knockout. In contrast, 7 days after transfection (at 7 DIV), N-cadherin protein was almost completely absent in CreEGFP+EGFP expressing neurons (Fig. S2B, C), whereas EGFP expressing control neurons showed strong N-cadherin expression. These results indicate that N-cadherin protein can be maintained after Cre-induced gene knockout for only a few days.

**Figure 4:**
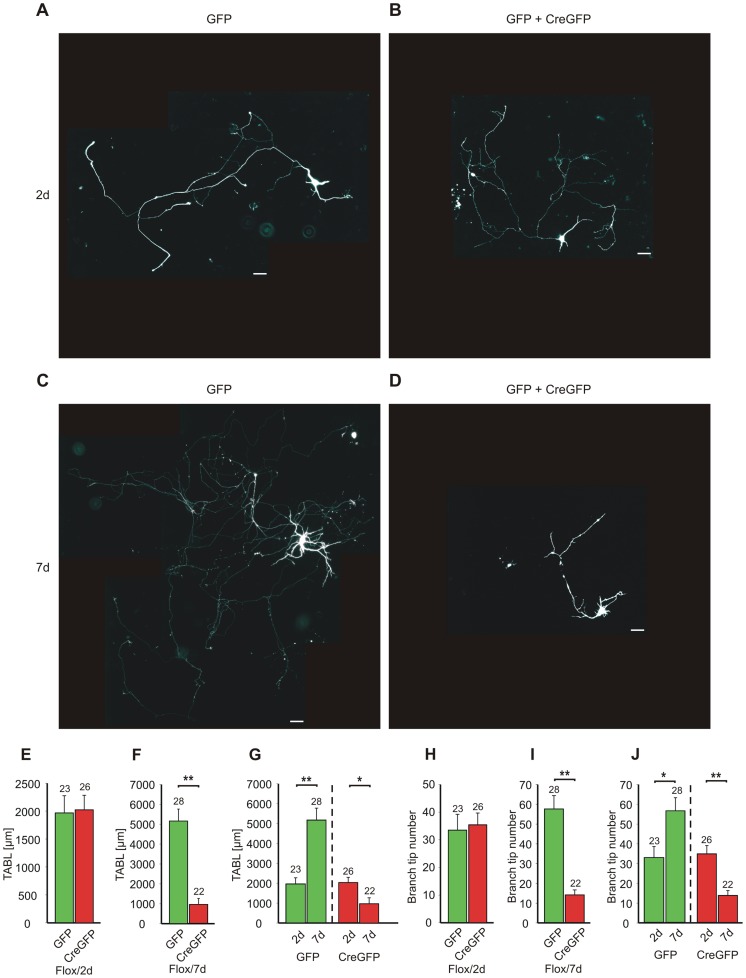
Long-term asymmetric expression of N-cadherin induces axon retraction. (**A–D**) Cultured neurons from floxed N-cadherin mice were cotransfected with creEGFP + EGFP to induce a conditional N-cadherin knockout in individual neurons. Reconstructions of whole cells from EGFP fluorescence images including the axon (composite images from several overlapping original images on black background) are shown. Neurons transfected with either EGFP (GFP, control; A, C) or creEGFP+EGFP (GFP+Cre-GFP, conditional knockout; B, D) at 2 days (2d, 7 DIV) and at 7 days (7d, 14 DIV) after transfection at 5 DIV and at 7 DIV, respectively, are shown. Note that neurons in A, B represent the morphological stage (7 DIV) at which neurons in C, D were transfected. Scale bars: 10 µm. (**E–J**) Quantification of axon length and branching. (**E, F**) Total axonal branch length (TABL) 2 days (E) and 7 days (F) after transfection. Note the strongly reduced axon length after long-term asymmetric expression of N-cadherin. (**G**) Comparison of TABL at 2d and 7d. Control neurons show axon growth, whereas conditional N-cadherin knockout neurons show axon retraction. (**H–J**) Branch tip number at 2 days (H) and at 7 days after transfection (I), and comparison between 2d and 7d (J). Means ± SEM. *n* (cells) is indicated on bars. *, *P*≤0.01; **, *P*<0.001 Students t-test.

We next analysed in individual EGFP expressing neurons (Fig. 4A–D) basic morphological parameters of the axon such as the total length of axonal branches and the number of branch tips. As expected from the presence of N-cadherin protein in all transfected neurons (Fig. S2A), 2 days after transfection (at 5 DIV), neither the total axonal branch length (TABL) nor the number of axonal branch tips differed significantly between creEGFP transfected and control neurons (transfected with EGFP only; Fig. 4A, B, E, H). Total dendritic branch length was also not affected (Fig. S3A). Strikingly, 7 days after transfection (at 7 DIV) – after N-cadherin had disappeared in creEGFP transfected neurons (Fig. S2B) – TABL and the number of axonal branch tips were strongly (*P*<0.001) reduced in N-cadherin knockout neurons (Fig. 4C, D, F, I). Because transfection was done at 7 DIV in the latter experiment, the morphology of the neurons studied at 7 DIV (transfected at 5 DIV) can be used as a starting point for the analysis of relative changes in axon morphology between 7 DIV and 14 DIV (Fig. 4G, J). As expected, in control EGFP expressing cells this analysis indicated a strong axon growth between 7 DIV and 14 DIV. In sharp contrast, in creEGFP + EGFP expressing neurons the comparison between 7 DIV and 14 DIV cells revealed clear axon retraction. Compared to 7 DIV (2 days after transfection), both TABL and the number of axonal branch tips were strongly diminished in N-cadherin knockout neurons at 14 DIV (7 days after transfection; Fig. 4G, J). In addition, also the growth of dendrites was inhibited in N-cadherin knockout neurons between 7 DIV and 14 DIV (Fig. S3). Expression of creEGFP + EGFP in cultured cortical neurons from non-floxed, wildtype mice did not alter TABL, the number of axonal branch tips, and dendrite growth 7 days after transfection (at 7 DIV) (Fig. S4). In summary, our results clearly indicate that long-term asymmetric expression of N-cadherin induces both synapse elimination and axon retraction.

### Asymmetric expression of N-cadherin in the cortico-tectal projection during in vivo development

**Figure 5:**
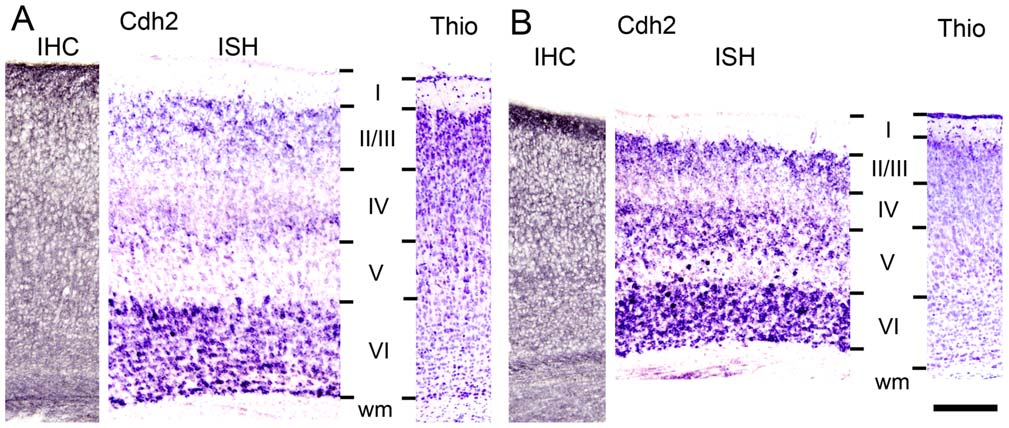
*In vivo* expression of N-cadherin in the cerebral cortex during postnatal mouse development. (**A, B**) Immunohistochemistry (IHC) and in situ hybridization (ISH) to detect expression of N-cadherin (Cdh2) at the protein level and mRNA level, respectively. Coronal sections of the somatosensory cortex (A) and the visual cortex (B) of the mouse at postnatal day 6. Note the almost complete lack of N-cadherin mRNA expression in layer V somatosensory neurons (A, ISH) and the relatively strong expression in a subpopulation of layer V visual neurons (B, ISH). IHC did not show layer-specific expression, because pyramidal cell dendrites extend over several layers. For identification of cortical layers, a corresponding Nissl stain (Thio) is shown in the right panels. Other abbreviations: I–VI, cortical layers I–VI; wm, white matter. Scale bar: 200 µm (for A, B).

To study whether an asymmetric expression of N-cadherin occurs during in vivo development, we analysed N-cadherin expression in different areas of the mouse neocortex using in situ hybridization (ISH) and immunohistochemistry (IHC). In the developing somatosensory cortex at P6, a layer-specific mRNA expression pattern was evident (Fig. 5A). Interestingly, N-cadherin mRNA expression was almost completely absent in layer V, whereas expression was found in the neighbouring layers IV and VI. Layers II/III also exhibited N-cadherin mRNA expression. Similar expression patterns have been described previously [44], [45]. In contrast, in the developing visual cortex at P6, N-cadherin mRNA expression was more prominent in layer V with rather strong expression in a subpopulation of neurons (Fig. 5B).

**Figure 6:**
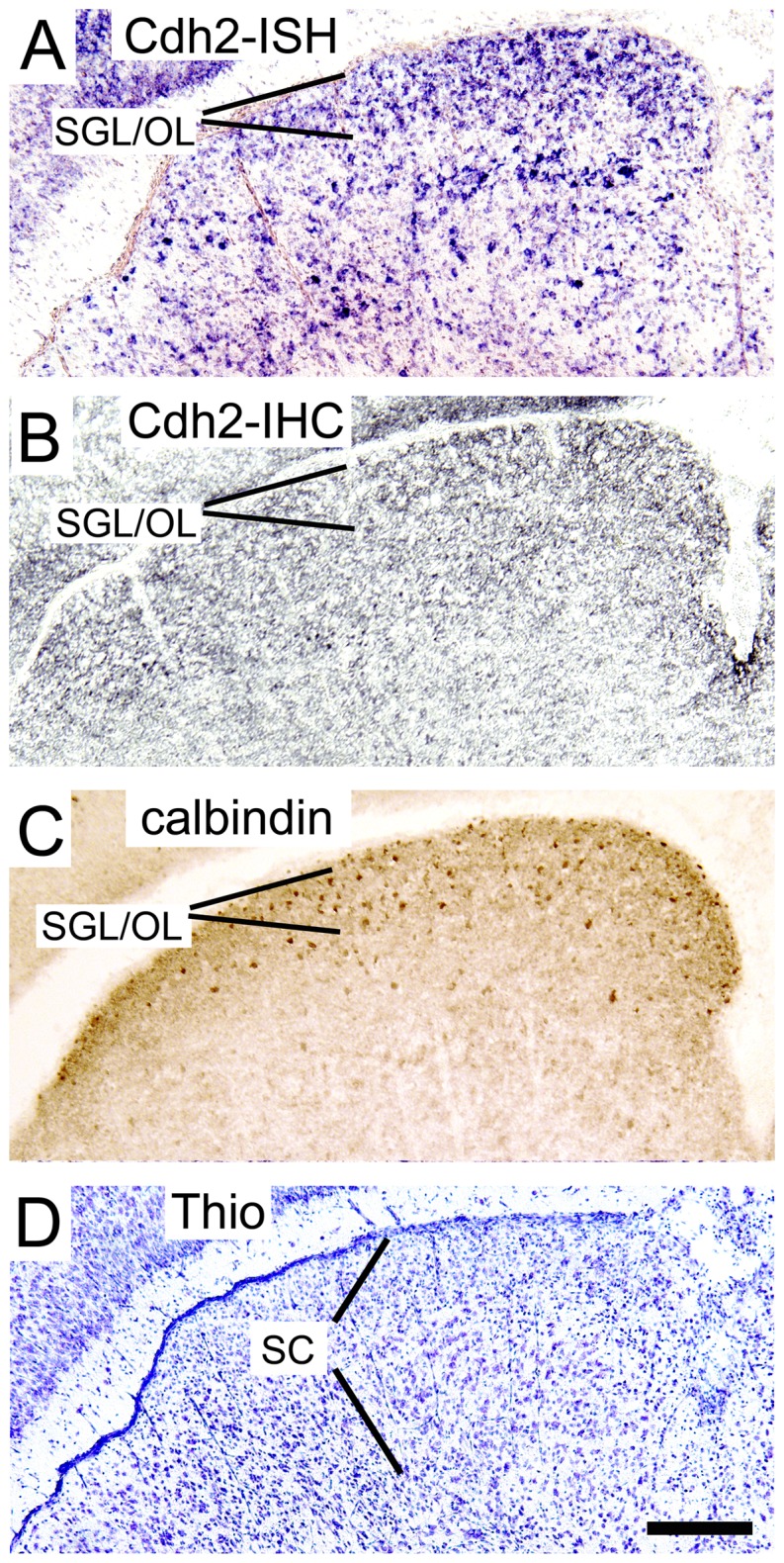
*In vivo* expression of N-cadherin in the superior colliculus (**SC**) **during postnatal mouse development.** (**A**) In situ hybridization (ISH) to detect N-cadherin (Cdh2) mRNA expression. Note the strong expression of N-cadherin in neurons of the superficial collicular layers (prospective superficial gray layer [SGL] and optic layer [OL]) where most visual cortical axons terminate. (**B, C**) Immunohistochemistry (IHC) to detect N-cadherin protein (B) and calbindin D28k protein (C), a marker for SGL and OL. (**D**) Nissl stain of a section adjacent to that shown in A and B. Coronal sections through the postnatal day 6 superior colliculus are shown. Scale bar: 200 µm (for A–D).

The projection of layer V pyramidal neurons of the somatosensory cortex to the colliculus superior is well known to be eliminated in rodents during the second postnatal week (P6-P14]) [46], [47]. We therefore also studied the expression of N-cadherin in the colliculus superior by ISH and IHC (Fig. 6). Strikingly, at P6 we found a strong expression of N-cadherin mRNA in the superficial layers (Fig. 6A) where cortical axon collaterals have been described to terminate [48]. Immunohistochemistry confirmed the expression of N-cadherin in the colliculus superior at the protein level (Fig. 6B). To visualize the superficial layers, we performed IHC for calbindin D28k, a marker for the superficial gray layer (SGL) and optic layer (OL) in the adult colliculus superior (Fig. 6C; [49]). Thus, an asymmetric expression of N-cadherin appeared to exist in the cortico-collicular projection in the somatosensory system, which might at least in part underlie the developmental elimination of this projection. In the visual system, the cortico-collicular projection of layer V pyramidal neurons is not eliminated [46], [47]. In line with this, we found N-cadherin expression at least in a subpopulation of layer V neurons in the developing visual cortex.

## Discussion

In this paper, we present evidence that an asymmetric, selectively postsynaptic expression of the transmembrane adhesion molecule N-cadherin induces an impairment of synaptic function at central excitatory synapses. Furthermore, this functional defect was – with clear delay – accompanied by an elimination of glutamatergic synapses. In addition, a retraction of axonal branches was observed in cortical neurons upon loss of N-cadherin selectively in the presynaptic cell. This sequence of events is in line with synapse elimination at the neuromuscular junction, where functional imbalances precede synapse elimination and axon retraction [1], [50], [51]. *In vivo*, asymmetric expression of N-cadherin was found to occur during the postnatal development of the cortico-tectal projection in the somatosensory system. In line with our findings *in vitro*, axon collateral retraction is well known in this developmentally transient projection [46], [47].

We studied the consequences of asymmetric N-cadherin expression in two closely related cell culture systems, mouse ES cell derived neurons and primary cultured cortical neurons. Using a retinoic acid induced *in vitro* differentiation within embryoid bodies, we [28], [37] and others [38] have shown that ES cell derived neurons exhibit functional properties similar to primary cultured cortical neurons, i.e. electrical excitability, and functional glutamatergic and GABAergic synapses exhibiting characteristic PSCs. A direct comparison of ES cell derived neurons and primary cultured cortical neurons (Jüngling and Gottmann, unpublished data) revealed a similar dendritic synapsin-I puncta density (at 9–10 DIV), and a tendency to reduced frequencies and amplitudes of AMPA mEPSCs (at 11–13 DIV) in ES cell derived neurons.

Upon asymmetric, selectively postsynaptic expression of N-cadherin, we found a strongly reduced frequency of AMPA mEPSCs, whereas the amplitudes of AMPA mEPSCs were not significantly altered. Because the number of synapses was unaffected on a short-term time scale, this indicates that a presynaptic functional impairment in vesicle release was induced by asymmetric N-cadherin expression. The functional defect was accompanied by a compensatory increase in the size of presynaptic vesicle clusters. This is in line with previous studies demonstrating that synaptic inactivity leads to a compensatory increase in presynaptic vesicle pool size [40], [41]. Our present findings on AMPA mEPSCs are further supported by previous paired patch-clamp recordings in chimeric cocultures of N-cadherin knockout, ES cell derived neurons and wildtype cortical neurons [28]. In these chimeric cocultures, a mis-match situation with presynaptic expression and postsynaptic knockout of N-cadherin in mature neurons (not studied in the present paper) had a relatively mild effect on vesicle replenishment exclusively during strong repetitive synaptic activity. This effect was similar to the defect observed in overall N-cadherin knockout synapses [28]. In stark contrast, synaptic pairs with presynaptic knockout and postsynaptic expression of N-cadherin (same mis-match as studied in the present paper with mEPSCs) exhibited strongly reduced single action potential-evoked AMPA EPSCs, thus not requiring repetitive stimulation to observe a functional defect [28]. Strongly confirming our AMPA mEPSC data, a presynaptic functional defect was indicated by an increased failure rate of evoked AMPA EPSCs [28]. Analogous mis-match expression experiments have been described for the cell adhesion molecule NCAM in cultured hippocampal neurons [52]. In this work, the postsynaptic absence of NCAM led to reduced amplitudes of evoked AMPA EPSCs and to a reduced number of presynaptic sites, whereas the selectively presynaptic absence of NCAM did not induce any changes [52]. Although the exact effects of asymmetric expression of NCAM are rather different to what we describe in this paper for N-cadherin, these previous and our recent findings are in line with the idea that mis-match expression of synaptic adhesion molecules leads to functional synaptic defects and subsequently to the elimination of synapses.

On a more long-term time scale, asymmetric N-cadherin expression led to a clear reduction in the number of presynaptic sites. However, during the major phase of synapse formation within the first two weeks in culture, this might indicate a slow down of synaptogenesis as well as a real elimination of already formed synapses. To confirm synapse elimination, we studied asymmetric N-cadherin expression at a later stage in culture and observed a substantial loss of both pre- and postsynaptic sites, and most importantly a loss of *bona fide* synapses identified by colocalization of pre- and postsynaptic markers. Although to a relatively low extent (only about 40% of neurons), also autapses formed in our cultures. As expected, these autapses were not affected by N-cadherin expression, because they are able to express N-cadherin both pre- and postsynaptically. We further observed a strong trend to an almost complete elimination of autapses with prolonged time in culture. This elimination process however, appeared to be based on different mechanisms, because it also occurred without any N-cadherin expression.

We further observed a strong loss of axonal branches upon asymmetric N-cadherin expression in cultured cortical neurons that was induced by a conditional knockout of N-cadherin in individual neurons. Related to our results, an impaired axon/neurite outgrowth on N-cadherin substrate upon expression of a dominant negative, extracellularly truncated N-cadherin construct was observed in individual hippocampal neurons in culture [14], [53]. Because our neurons were cultured together with glial cells representing a potential N-cadherin expressing cellular substrate for axon outgrowth [54], [55], an altered neuron-astrocyte interaction might lead to a reduced growth of axons. However, because we found a clear retraction of axonal processes instead of just a reduced axon outgrowth, a mechanism based on synaptic interactions with postsynaptic neurons is likely to be involved in addition. In parallel to axon retraction, we also observed a loss of dendrite growth upon conditional knockout of N-cadherin in individual neurons. This effect of the absence of N-cadherin in the postsynaptic cell is in line with previous studies showing that dendrite growth in hippocampal neurons strongly depends on the function of the N-cadherin/catenin complex [56], [57].

Regarding the molecular mechanisms involved in the effects of asymmetric N-cadherin expression, there are two principal possibilities: i.) a relatively unspecific distortion of the stabilizing nanostructures at the synapse and ii.) a highly specific *cis*- or *trans*-interaction with a N-cadherin associated receptor that activates a signaling pathway. Synapses are thought to be structurally stabilized by a molecularly complex system of several transsynaptically interacting adhesion molecules [2], [10], [58]. N-cadherin might be of particular importance for synapse maintenance, because of its well known homophilic interactions both in *trans*- and *cis*-configuration resulting in a highly organized structural component of the synapse [24], [59]. N-cadherin molecules that cannot interact with transsynaptic counterparts might therefore strongly distort the synaptic adhesion system leading to destabilization and elimination of synapses. In line with this idea, overexpression of C-terminal fragments of N-cadherin unable to interact homophilically has been described to destabilize glutamatergic synapses in hippocampal and cortical neurons [11], [14], [18], [60]. Alternatively, postsynaptic N-cadherin not binding transynaptically to presynaptic N-cadherin might trigger a yet unknown, but highly specific signaling pathway that retrogradely impairs presynaptic function. There are well described examples of *cis*-interacting N-cadherin associated receptors, such as the FGF receptor [7], [61], the protocadherin Arcadlin [62], [63], the Slit-receptor Roundabout [64], [65], and other transmembrane receptors recruited to the N-cadherin/catenin complex [e.g. 66]. N-cadherin has further been described to interact with the N-terminal domain of GluR2 resulting in GluR2 recruitment and a GluR2-mediated increase in mEPSC frequency [39]. In line with N-cadherin induced AMPA receptor recruitment, we also observed a slight trend to increased AMPA mEPSC amplitudes upon postsynaptic N-cadherin mis-match expression. However, in respect to AMPA mEPSC frequency we found the opposite effect (reduction) than would be expected from GluR2 recruitment (enhancement). Thus, other mechanisms appear to be dominating in our strong N-cadherin mis-match expression situation. Presynaptic N-cadherin binding proteins, which are able to interact in *trans* across the synaptic cleft and are not cadherins have not yet been characterized. Nevertheless, specific transsynaptic signaling mechanisms might be activated by postsynaptic N-cadherin leading to synapse dysfunction and synapse elimination.

Our *in vivo* analysis of N-cadherin expression using in situ hybridization and immunohistochemistry further indicated an asymmetric, selectively postsynaptic expression of N-cadherin in the cortico-tectal projection of the developing mouse somatosensory system. In line with our *in vitro* experiments, this projection is developmentally transient and gets eliminated [46], [47]. Such elimination processes of non-matching (in respect to a specific cadherin) connections might at least in part underlie the long-standing observation that the expression of specific cadherins in functionally connected brain areas is well corresponding [30], [31], [32]. Recently, the importance of matching cadherin expression for synapse formation and maintenance has also been demonstrated for cadherin-6 in retinal ganglion cells [33] and for cadherin-9 in hippocampal neurons [34].

In conclusion, we found that an asymmetric, selectively postsynaptic expression of N-cadherin at central glutamatergic synapses appears to initiate a sequence of synaptic dysfunction, synapse elimination, and retraction of axonal branches. Similar events are well known to underlie the developmental maturation of innervation patterns at the neuromuscular junction [1], [50], [51]. Our results further strengthen the idea that – in addition to selective formation of synapses – the elimination of molecularly inappropriate synapses is of major importance for the establishment of complex, highly specific neuronal circuits [4].

## Materials and Methods

### Ethics statement

No ethical approval was required, because experiments were performed on primary cultured mouse cells, and on neurons derived from mouse cell lines. Also for the in situ hybridization and immunhistochemistry experiments performed on mouse brain sections no ethical approval was required, because no experimentation on living animals was done.

### Cell culture, *in vitro* differentiation of ES cells, transfection and aggregation assay

Homozygous N-cadherin knockout, mouse ES cells were provided by Dr. R. Kemler, Max-Planck-Institute for Immunobiology, Freiburg, Germany [35]. The mouse ES cell line LF2 (wildtype ES cells) was obtained from Dr. I. Chambers, Institute for Stem Cell Research, Edinburgh, UK [67].

Homozygous N-cadherin knockout, mouse ES cells [35], [68] were cultivated on mouse embryonic feeder cells using a standard protocol. *In vitro* differentiation of ES cells occurred upon formation of embryoid bodies. ES cell derived neurons were purified by immunopanning with L1 antibodies and further cultured on glial feeder cells [18], [37]. Foetal cortical neurons were obtained from wildtype C57BL/6 mice and from homozygous floxed N-cadherin mice [42] and were cultured using a standard protocol [18], [36]. ES cell derived neurons and cultured cortical neurons were transfected using Lipofectamine 2000 (Invitrogen) according to the manufactureŕs instructions [69]. The following plasmids were used: pEGFP-N1 (EGFP, Clontech); pMS149.1 (full-length mouse N-cadherin, provided by Dr. R. Kemler); pBS598 EF1alpha-EGFPcre (creEGFP, addgene, from Dr. B. Sauer); pDsRed2-VAMP2 (provided by Dr. T. Dresbach); pGW1 PSD95-EGFP, provided by Dr. D. Bredt).

To functionally check the N-cadherin construct, a cell aggregation assay was performed with CHO cells that were transfected with N-Cadherin vector or water control using jetPEI (Peqlab, Erlangen, Germany). 24 h after transfection cells were dispersed to a single cell solution by trypsinization with 0,125% trypsin in EBSS (Ca^2+^ and Mg^2+^ free) plus 5 mM CaCl_2_ for 7 min. Trypsin was neutralized with 10% FBS and cells were washed once with EBSS plus 5 mM CaCl_2_. Then cells were resuspended to 2,5×10^5^/ml in EBSS containing 0.2% BSA in either the presence or absence of 5 mM CaCl_2_. For aggregation cells were added to a 24-well TC-plate (0.5 ml/well) and incubated for 1 hr at 37°C. During this time the plate was gently shaked every 15 min. Aggregate formation was observed with a Zeiss PrimoVert Microscope, using a 10× objective.

### Electrophysiology and data analysis

Whole-cell patch-clamp recordings were performed using standard conditions [28] at a holding potential of −60 mV. Patch pipette solution contained 110 mM KCl, 0,25 mM CaCl_2_, 10 mM EGTA, 20 mM Hepes, pH = 7.3. Extracellular solution contained 130 mM NaCl, 5 mM KCl, 1 mM MgCl_2_, 2,5 mM CaCl_2_, 20 mM Hepes, pH = 7.3. AMPA receptor mediated miniature EPSCs were isolated by addition of 1 µM TTX and 10 µM gabazine to the extracellular solution. AMPA mEPSCs were completely blocked by addition of 10 µM DNQX. Autaptic AMPA receptor mediated EPSCs were elicited by triggering action potentials with short (1ms) depolarizing pulses and were recorded in the presence of elevated extracellular Ca^2+^ (5 mM) and 10 µM gabazine at a holding potential of −60 mV. About 1–3 cells were recorded per culture dish. Postsynaptic currents were quantitatively analysed using Mini Analysis software (Synaptosoft) and pCLAMP 9 software (Molecular Devices).

### Immunocytochemistry, fluorescence imaging and data analysis

Immunocytochemical stainings for VAMP2 and N-cadherin were performed using a standard protocol [18]. For N-cadherin stainings, we partially used sandwich cultures enabling separation of neurons and glial cells. Primary antibodies used were mouse-anti-VAMP2 (Synaptic Systems), rabbit anti-N-cadherin (Abcam; antibody against C-terminal region), and mouse anti-N-cadherin (BD Biosciences). Fluorescence imaging was performed using an inverted Axiovert 200M deconvolution microscope (Zeiss) in combination with a CoolSNAP ES CCD camera (Photometrics) [18]. Deconvolution of original images was performed using AutoDeblur software (Visitron Systems) and maximum intensity images were calculated from z-stacks using MetaMorph software (Visitron Systems). Further digital image processing was performed by thesholding and low-pass filtering the image prior to quantitatively analysing synaptic puncta. About 2–5 neurons were analysed per culture dish. Morphometric analysis of EGFP expressing neurons was done using MetaVue software (Visitron Systems). To determine total axonal branch length, individual axons were traced from their endpoints back to a single small caliber process at the cell soma. About 1–3 neurons were analysed per culture dish.

### N-cadherin in situ hybridization and immunostaining of sections

Antisense and sense cRNA probes were labeled with digoxigenin by *in vitro* translation with the bMN3sk+ vector that contains a 980-bp fragment of the extracellular domain (EC1–EC3) of the mouse N-cadherin cDNA (gift of Dr. M. Takeichi). Frontal brain sections were obtained from postnatal day 6 (P6) mouse pups killed by rapid decapitation. No ethical approval was required according to national guidelines for the care of animals. For in situ hybridization, sections were fixed in formaldehyde solution (4% w/v in phosphate-buffered saline [PBS]) on ice, following a previously published protocol [31]. After treatment with 1 mg/ml Proteinase K (Merck) in 100 mM Tris (pH 8), 50 mM EDTA at 37°C for 1–3 min, the sections were hybridized for 20 h at 55°C in a humid chamber with sense or antisense cRNA probes, respectively, in hybridization buffer. The hybridization was followed by extensive washes and RNase A treatment (20 mg/ml in 10 mM Tris, 1 mM EDTA, 0.5 M NaCl, pH 8.0) and by incubation with alkaline phosphatase-conjugated Fab fragments against digoxigenin (Boehringer) overnight at 4°C. Sections were reacted with X-phosphate (50 mg/ml) and nitroblue tetrazolium salt (25 mg/ml) in alkaline buffer and mounted in Entellan (Merck).

For immunohistochemistry, adjacent sections were stained with rat monoclonal antibody against mouse N-cadherin (clone MNCD2, kind gift of Dr. M. Takeichi; [70]). To visualize the optic layers of the superior colliculus, frontal P6 sections were stained with polyclonal rabbit antiserum against rat recombinant calbindin D-28k (Swant, clone CB38a, dilution 1:1000). For immunostaining, a previously described protocol was followed [31]. For neuroanatomical orientation, adjacent sections were counterstained for Nissl substance with thionin [71]. Photomicrographs of the sections were taken.

### Statistical analysis

Statistical significance was determined by using Students t-test (or ANOVA, if more than two data sets were compared).

## Supporting Information

Figure S1 **Expression of N-cadherin in N-cadherin knockout neurons and CHO cells.** (**A**) Western blot demonstrating the absence of N-cadherin expression in N-cadherin knockout cells. Embryoid bodies formed by differentiating control (+/+) and homozygous N-cadherin knockout (−/−) ES cells were used for Western. Actin was used as loading control. (**B**) Functionality of the N-cadherin expression vector was tested by using a CHO cell aggregation assay. *Left:* CHO cells transfected with N-cadherin (Ncad) and control (Mock) CHO cells. 24 hours after transfection. *Center:* N-cadherin transfected CHO cells and control CHO cells after a 60 min reaggregation period in the presence of 5 mM Ca^2+^. *Right:* 60 min reaggregation in the absence of extracellular Ca^2+^. Note the enhanced Ca^2+^-dependent aggregation of CHO cells transfected with the N-cadherin vector. Scale bars: 100µm. (**C**) Quantification of the dendritic density of immuncytochemically stained N-cadherin puncta in cultured wildtype cortical neurons (12 DIV) and in N-cadherin knockout neurons expressing N-cadherin (transfected at 12 DIV, 8 days after transfection). (**D**) Expression of N-cadherin+EGFP (Ncad) in N-cadherin knockout neurons (see also Fig. 1A, B) did not affect dendrite growth as compared to control EGFP expression (GFP). TDBL: total dendritic branch length. 11–13 DIV, 2 days after transfection. Means ± SEM. *n* (cells; in (D) dendrites) is indicated on bars. *, *P*<0.01 Students t-test.
(TIF)

Figure S2 **Confirmation of the loss of N-cadherin upon CreEGFP expression in individual cultured neurons from floxed N-cadherin mice** (**conditional knockout**)**.** (**A, B**) N-cadherin expression in individual neurons 2 days after creEGFP transfection (at 5 DIV) (A) and 7 days after creEGFP transfection (at 7 DIV) (B). *Upper panels:* Control neurons transfected with EGFP only (GFP control). *Lower panels:* Neurons cotransfected with creEGFP + EGFP (GFP+CreGFP). *Left images:* EGFP fluorescence (green). *Center images:* N-cadherin immunofluorescence (red). *Right images:* merged fluorescence images (overlay). Scale bars: 10 µm. Note the loss of N-cadherin expression 7 days after transfection with creEGFP (B, lower panel). *n* (transfected cells) for (A): 14, 25, and for (B): 28, 26 (stained with either rabbit or mouse anti-N-cadherin). (**C**) Quantification of N-cadherin immunofluorescence in transfected neurons. Fluorescence intensity was determined at the cell soma and background signal was subtracted. Only cells stained with mouse anti-N-cadherin antibody were included, because the rabbit anti-N-cadherin antibody exhibited a relatively high non-specific fluorescence at the cell soma. Means ± SEM. *n* (cells) is indicated on bars. **, *P*<0.001, Students t-test.
(TIF)

Figure S3 **Conditional knockout of N-cadherin inhibits dendrite growth.** (**A–C**) Quantitative analysis of dendrites in conditional N-cadherin knockout neurons 2 days (Flox/2d) and 7 days (Flox/7d) after transfection with creEGFP + EGFP (CreGFP). EGFP expression (GFP) was used as control. Same cells as in Fig. 3. (**A, B**) Total dendritic branch length (TDBL) at 2d (A) and at 7d (B) after transfection. (**C**) Comparison of TDBL between 2d and 7d after transfection (7 DIV versus 14 DIV) revealed significant dendrite growth in EGFP expressing control neurons, whereas TDBL was slightly reduced in conditional N-cadherin knockout neurons. Means ± SEM. *n* (cells) is indicated on bars. *, *P*<0.05; **, *P*<0.01 Students t-test.
(TIF)

Figure S4 **Transfection with the creEGFP vector does not affect axons and dendrites in cultured wildtype cortical neurons.** (**A–C**) Quantitative analysis of axons and dendrites in wildtype cortical neurons 7 days (Non-flox/7d) after transfection (at 7DIV) with creEGFP + EGFP (CreGFP). EGFP expression (GFP) was used as control. Neither total axonal branch length (TABL, A) nor axon branch tip number (B) nor total dendritic branch length (TDBL, C) were affected by expression of CreEGFP in wildtype cortical neurons. Means ± SEM. *n* (cells) is indicated on bars. Students t-test.
(TIF)
