# Supplementary figures and images for: Asymmetric N-Cadherin Expression Results in Synapse Dysfunction, Synapse Elimination, and Axon Retraction in Cultured Mouse Neurons
Source: PLoS One. 2013 Jan 31;8(1):e54105. doi: 10.1371/journal.pone.0054105 (PMC3561303; doi:10.1371/journal.pone.0054105)

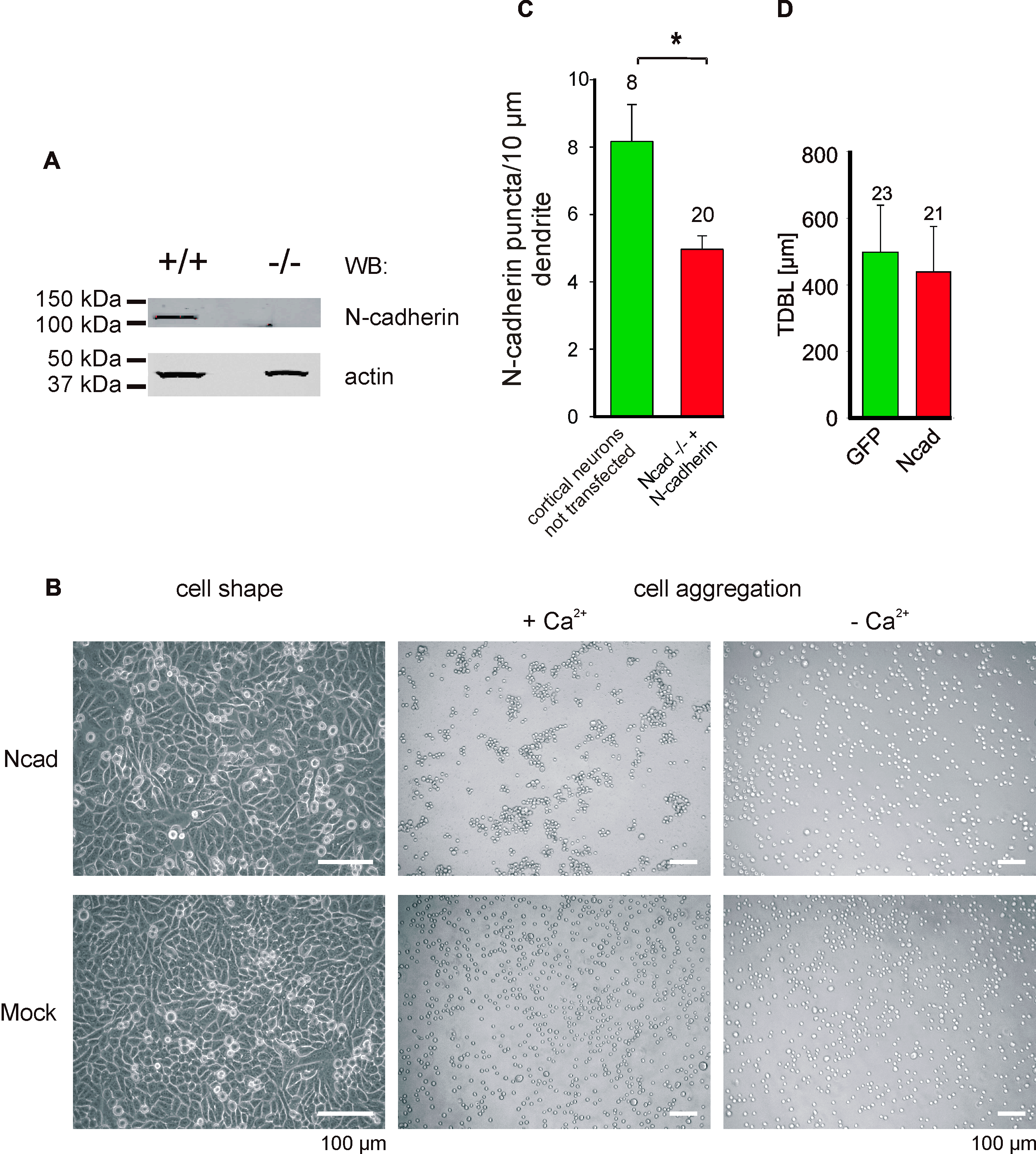

Supplement: Figure S1 — Expression of N-cadherin in N-cadherin knockout neurons and CHO cells. (A) Western blot demonstrating the absence of N-cadherin expression in N-cadherin knockout cells. Embryoid bodies formed by differentiating control (+/+) and homozygous N-cadherin knockout (−/−) ES cells were used for Western. Actin was used as loading control. (B) Functionality of the N-cadherin expression vector was tested by using a CHO cell aggregation assay. Left: CHO cells transfected with N-cadherin (Ncad) and control (Mock) CHO cells. 24 hours after transfection. Center: N-cadherin transfected CHO cells and control CHO cells after a 60 min reaggregation period in the presence of 5 mM Ca2+. Right: 60 min reaggregation in the absence of extracellular Ca2+. Note the enhanced Ca2+-dependent aggregation of CHO cells transfected with the N-cadherin vector. Scale bars: 100µm. (C) Quantification of the dendritic density of immuncytochemically stained N-cadherin puncta in cultured wildtype cortical neurons (12 DIV) and in N-cadherin knockout neurons expressing N-cadherin (transfected at 12 DIV, 8 days after transfection). (D) Expression of N-cadherin+EGFP (Ncad) in N-cadherin knockout neurons (see also Fig. 1A, B) did not affect dendrite growth as compared to control EGFP expression (GFP). TDBL: total dendritic branch length. 11–13 DIV, 2 days after transfection. Means ± SEM. n (cells; in (D) dendrites) is indicated on bars. *, P<0.01 Students t-test. (TIF) [file pone.0054105.s001.tif]

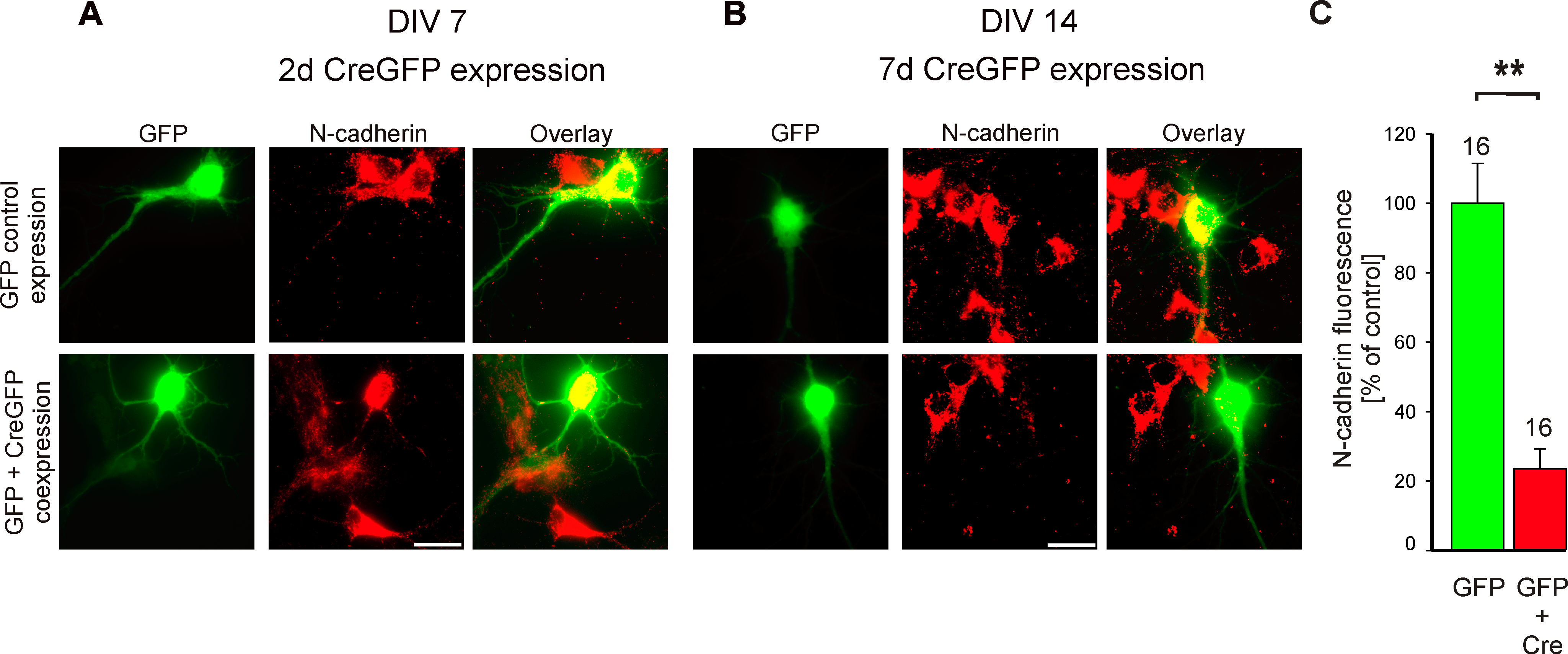

Supplement: Figure S2 — Confirmation of the loss of N-cadherin upon CreEGFP expression in individual cultured neurons from floxed N-cadherin mice (conditional knockout). (A, B) N-cadherin expression in individual neurons 2 days after creEGFP transfection (at 5 DIV) (A) and 7 days after creEGFP transfection (at 7 DIV) (B). Upper panels: Control neurons transfected with EGFP only (GFP control). Lower panels: Neurons cotransfected with creEGFP + EGFP (GFP+CreGFP). Left images: EGFP fluorescence (green). Center images: N-cadherin immunofluorescence (red). Right images: merged fluorescence images (overlay). Scale bars: 10 µm. Note the loss of N-cadherin expression 7 days after transfection with creEGFP (B, lower panel). n (transfected cells) for (A): 14, 25, and for (B): 28, 26 (stained with either rabbit or mouse anti-N-cadherin). (C) Quantification of N-cadherin immunofluorescence in transfected neurons. Fluorescence intensity was determined at the cell soma and background signal was subtracted. Only cells stained with mouse anti-N-cadherin antibody were included, because the rabbit anti-N-cadherin antibody exhibited a relatively high non-specific fluorescence at the cell soma. Means ± SEM. n (cells) is indicated on bars. **, P<0.001, Students t-test. (TIF) [file pone.0054105.s002.tif]

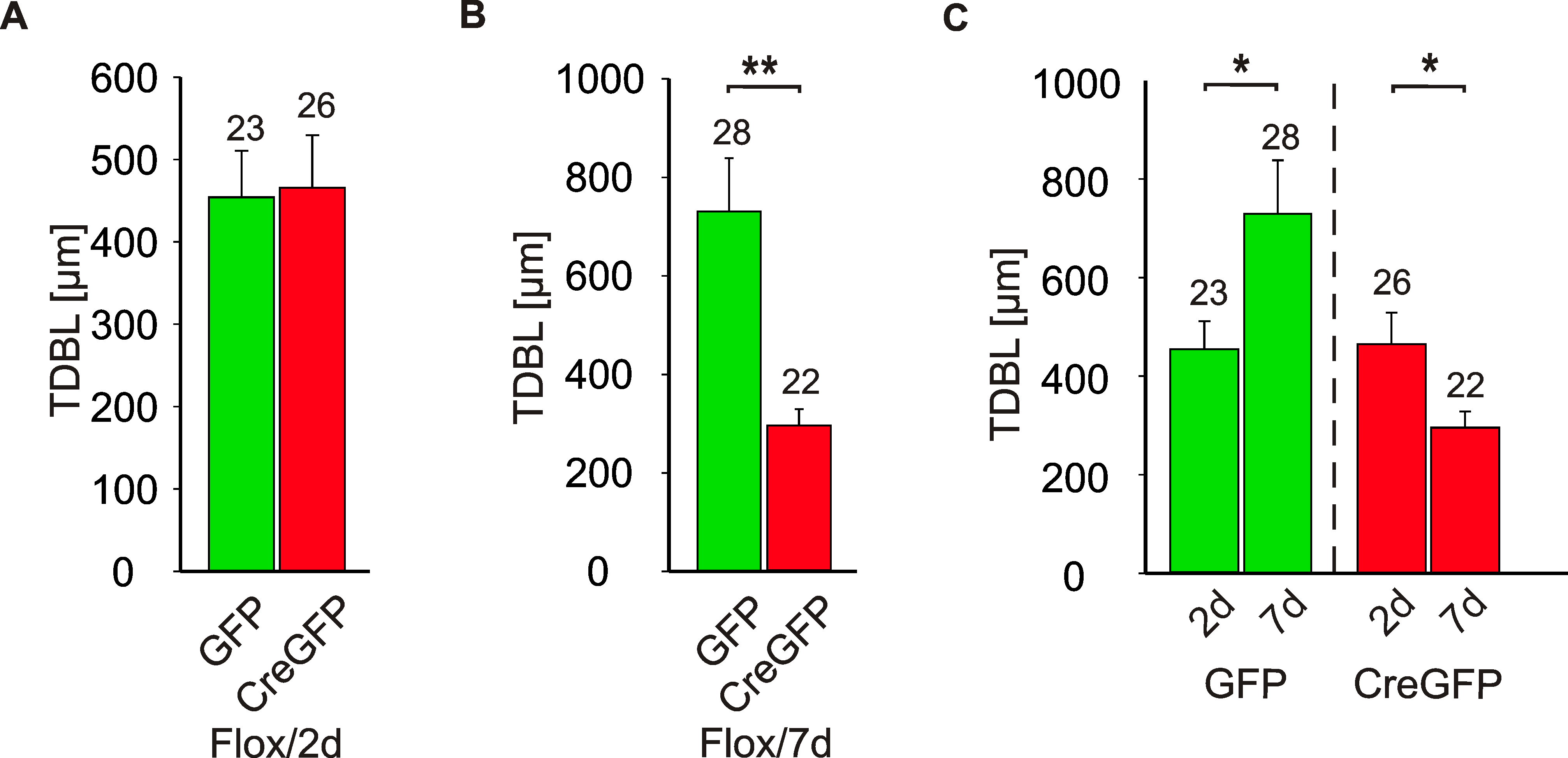

Supplement: Figure S3 — Conditional knockout of N-cadherin inhibits dendrite growth. (A–C) Quantitative analysis of dendrites in conditional N-cadherin knockout neurons 2 days (Flox/2d) and 7 days (Flox/7d) after transfection with creEGFP + EGFP (CreGFP). EGFP expression (GFP) was used as control. Same cells as in Fig. 3. (A, B) Total dendritic branch length (TDBL) at 2d (A) and at 7d (B) after transfection. (C) Comparison of TDBL between 2d and 7d after transfection (7 DIV versus 14 DIV) revealed significant dendrite growth in EGFP expressing control neurons, whereas TDBL was slightly reduced in conditional N-cadherin knockout neurons. Means ± SEM. n (cells) is indicated on bars. *, P<0.05; **, P<0.01 Students t-test. (TIF) [file pone.0054105.s003.tif]

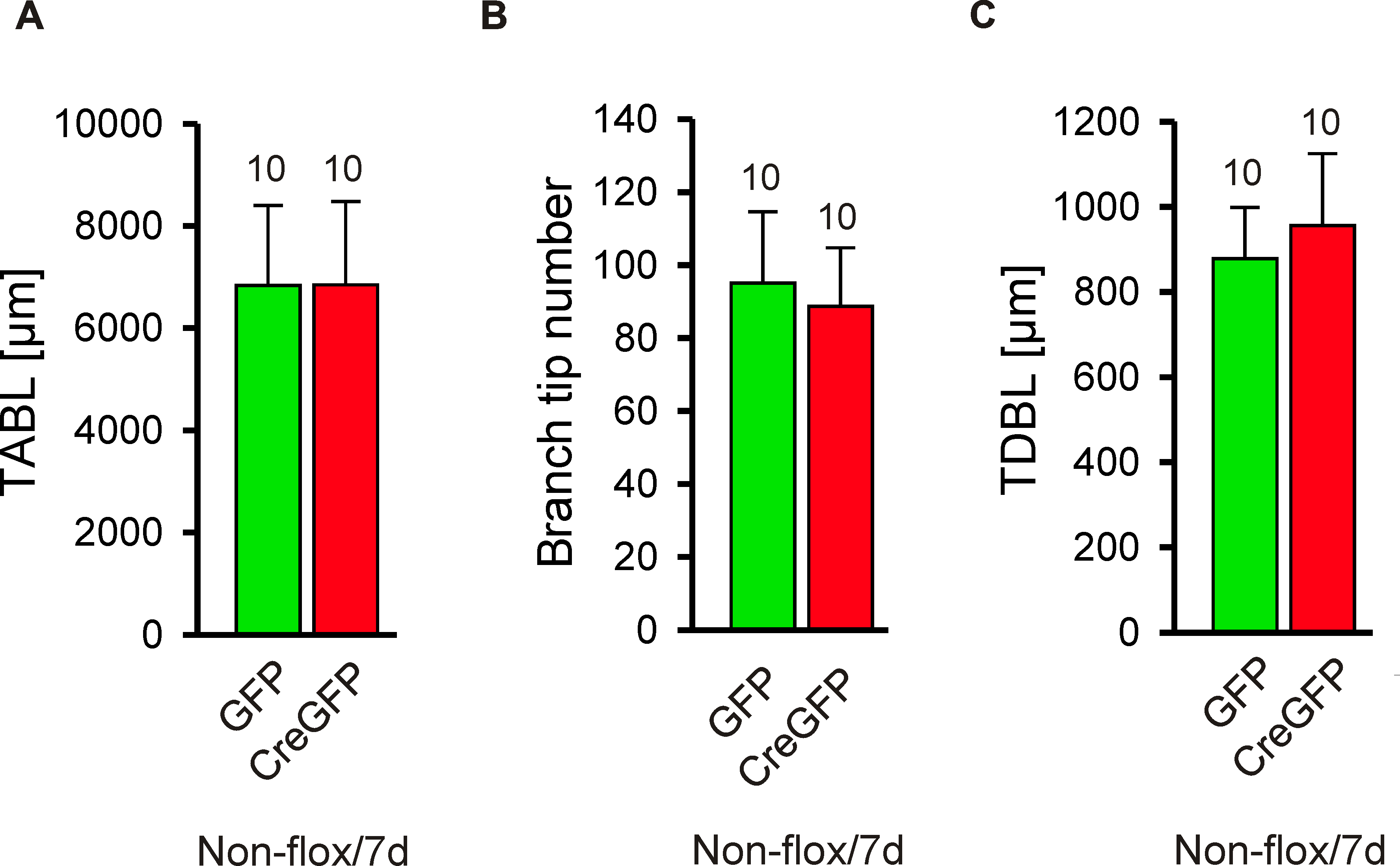

Supplement: Figure S4 — Transfection with the creEGFP vector does not affect axons and dendrites in cultured wildtype cortical neurons. (A–C) Quantitative analysis of axons and dendrites in wildtype cortical neurons 7 days (Non-flox/7d) after transfection (at 7DIV) with creEGFP + EGFP (CreGFP). EGFP expression (GFP) was used as control. Neither total axonal branch length (TABL, A) nor axon branch tip number (B) nor total dendritic branch length (TDBL, C) were affected by expression of CreEGFP in wildtype cortical neurons. Means ± SEM. n (cells) is indicated on bars. Students t-test. (TIF) [file pone.0054105.s004.tif]
